# Salmagundi

**Published:** 1886-01

**Authors:** 


### SALMAGUNDI.

EDITED BY E. G. BETTY, D. D. S.

M. PASTEUR is sixty-six years of age.

---

NO ONE knows the value of flowers who hasn't botany.—*Boston Post.*

---

WOMAN was made after man, and she has been after him ever since.—*Philadelphia Call.*

---

SPURGEON'S physical breaking down is said to be largely the result of over-indulgence in tobacco.

---

" BISMARCK is suffering the tortures of the damned, because he's ' Old-Man-Afraid-of-the-Dentist.' "

---

THE shortest and most significant obituary Vanderbilt received was from the *Buffalo Express* when it said: " A great railroad man has got his blue envelope."

---

SALMAGUNDI begins the third year with this number and returns thanks to her friends for their kindly help and hopes to have them call again ; call frequently.

---

A LONDON medical man says: " Be careful in your dealings with horse-radish. It irritates the stomach far more than spice, and an overdose will bring on an unpleasant sensation for days."

---

CANNON FARRAR says one of the responsibilities of this nation is to " strangle the growth of mammon worship." The dear, innocent old Cannon, how much did he take home with him? —*Binghampton Republican.*

Pompous physician (to patient's wife)—"Why did you delay sending for me until he was out of his mind?"

Wife—"Oh, doctor! while he was in his right mind, he wouldn't let me send for you."—*Clipping.*

---

J. M. Barton, M.D., of Philadelphia, reports in the *Medical and Surgical Reporter* for Nov. 7th, 1885, a number of interesting cases of fractures examined under the influence of nitrous oxide; he considers it superior to ether for the purpose.

---

Wasted Education.—It is proper that boys who smoke cigarettes should be expelled from public schools, as is being done in New Orleans. They are predestined for catarrh and consumption, and an education would be clearly wasted.—*Baltimore Herald.*

---

Some one placed a piece of limburger cheese in the lining of a Santa Cruz merchant's hat this week, and the merchant has been loudly proclaiming that the city needs a sewer system right away, as the smell of sewer gas was something awful.—*Santa Cruz* (Cal.) *Sentinel.*

---

Prompt Retort.—"What great blessing do we enjoy that the heathens know nothing about?" inquired a Sunday-school teacher.

"Soap!" was the answer that came out like the crack of a pistol from the small boy at the foot of the class.—*Chicago Ledger.*

---

The United States census for 1880 places the number of dentists in this country at 12,314; of these 61 are females. There are 2,637 males ranging in age from 16 to 59 years, and 54 females also of that class; 120 males are 60 years of age or over, one female also of this class. According to this there are about ten thousand male dentists of "uncertain age"; it's generally thought that this applies to women folks.

A case is related in a Dutch medical journal of paralysis of the facial muscles on one side, with loss of taste on that half of the tongue, following on a painful attack of herpes affecting the whole side of the face. It was noted that the eruption and the pain ceased on the supervention of the paralysis. The observer, Dr. Waller, considers that the branches of the fifth nerve only were affected.—*Medical and Surgical Reporter.*

---

Dr. Oscar H. Allis, of Philadelphia, tells of Dr. Gross: "That at the close of the last lecture of one of the sessions, he paused a moment, and glancing merrily toward his class, said: "I suppose you expect a parting word from me." Then, after a second pause, he said: "One of the most beautiful sights on earth is that of a young mother leading a little child. Gentlemen, marry early; cultivate flowers; occupy your minds with the delightful things of earth, and leave no time to the seductiveness of vice."

---

In the first generation a man reckons only two ancestors, his father and mother. In the second generation the two are changed into four, since he had two grandfathers and two grandmothers. Each of these four had two parents, and thus in the third generation there are found to be eight ancestors, that is, eight great-grandparents. In the fourth generation the number of ancestors is 16, in the fifth 32, in the sixth 64, in the seventh 128, in the tenth 1,024, in the twentieth 1,048,576, in the thirtieth 1,073,741,834. This may prove that all the world's akin.—*Chicago Living Church.*

---

London, December 25.—It will be remembered that Prof. Webster was convicted thirty-five years ago at Boston of the murder of Dr. Parkman, principally because the false teeth of the victim were found in Webster's furnace and were recognized by a dentist.

Criminal history repeats itself. In Mlle. Mercier's garden there was picked up a false tooth near where the supposed body of of Mlle. Menetret was found buried. A dentist here is positive that it is a tooth which he made for the dead woman.—Cable to *Commercial Gazette.*

---

RARE SYMPTONS OF LOCOMOTOR ATAXIA.--M. Charcot has recently had under his care, at the Salpetriere Hospital (*Journal de Medecine et de Chirurgie Pratique*), a man, aged fifty-two, who was suffering from locomotor ataxia. Besides the usual symptoms, the patient presented the peculiar deformity known as tabic foot. The right foot was broader and thicker than the left, and the arch much less marked. There was neither œdema nor pain. Professor Charcot is of opinion that the absence of pain and inflammation is a most important point in the diagnosis of all diseases of the joints caused by locomotor ataxy. M. Charcot's second case was that of a man with symptoms of tabes anæsthesia of the face; the loss of sensibility had begun in the upper lip, and invaded gradually the whole of the face, the mouth, and the soft palate; but the sense of taste was still retained. There were patches of anæsthesia on the chest, and of hyperæsthesia on the back. The patient presented, also, some remarkable trophic lesions; he had lost nine teeth without pain in one month, and at the same time small pieces of the inferior maxilla had become detached. The disease of the jaw showed, however, a decided tendency to spontaneous cure.

